# The Postbiotic Butyrate Mitigates Gut Mucosal Disruption Caused by Acute Ethanol Exposure

**DOI:** 10.3390/ijms25031665

**Published:** 2024-01-29

**Authors:** Mohamed Tausif Siddiqui, Yingchun Han, David Shapiro, Gail West, Claudio Fiocchi, Gail A. M. Cresci

**Affiliations:** 1 Department of Gastroenterology, Hepatology and Nutrition, Digestive Disease Institute, Cleveland Clinic, Cleveland, OH 44195, USA; siddiqm5@ccf.org (M.T.S.); fiocchc@ccf.org (C.F.); 2 Department of Inflammation & Immunity, Lerner Research Institute, Cleveland Clinic, Cleveland, OH 44195, USA; 3 Division of Pediatric Gastroenterology, Hepatology and Nutrition, Cleveland Clinic Children’s Hospital, Cleveland, OH 44195, USA

**Keywords:** acute ethanol, small intestine, ulcerative colitis, intestinal endothelium

## Abstract

We aimed to test how the postbiotic butyrate impacts select gut bacteria, small intestinal epithelial integrity, and microvascular endothelial activation during acute ethanol exposure in mice and primary human intestinal microvascular endothelial cells (HIMECs). Supplementation during an acute ethanol challenge with or without tributyrin, a butyrate prodrug, was delivered to C57BL/6 mice. A separate group of mice received 3 days of clindamycin prior to the acute ethanol challenge. Upon euthanasia, blood endotoxin, cecal bacteria, jejunal barrier integrity, and small intestinal lamina propria dendritic cells were assessed. HIMECs were tested for activation following exposure to ethanol ± lipopolysaccharide (LPS) and sodium butyrate. Tributyrin supplementation protected a butyrate-generating microbe during ethanol and antibiotic exposure. Tributyrin rescued ethanol-induced disruption in jejunal epithelial barrier, elevated plasma endotoxin, and increased mucosal vascular addressin cell-adhesion molecule-1 (MAdCAM-1) expression in intestinal microvascular endothelium. These protective effects of tributyrin coincided with a tolerogenic dendritic response in the intestinal lamina propria. Lastly, sodium butyrate pre- and co-treatment attenuated the direct effects of ethanol and LPS on MAdCAM-1 induction in the HIMECs from a patient with ulcerative colitis. Tributyrin supplementation protects small intestinal epithelial and microvascular barrier integrity and modulates microvascular endothelial activation and dendritic tolerizing function during a state of gut dysbiosis and acute ethanol challenge.

## 1. Introduction

Gut microbiota aid in digestion and metabolism, defend against pathogens, and support host immunity [1]. There are many factors, including antibiotics and alcohol exposure, that are known to cause gut dysbiosis, an imbalance in microbe abundance and disturbance in microbe metabolic activities [2]. In health, the microbiota remains in a homeostatic balance because of an intact intestinal barrier and other host-derived elements. In humans and animals, both acute and chronic ethanol exposure disrupt the intestinal barrier, resulting in endotoxin translocation into systemic circulation and inflammation and injury of various organs [3]. Intestinal endothelial cells line the microvasculature within the lamina propria of the intestinal mucosa. The intestinal endothelium provides a mechanical and functional (immune) barrier that may serve as a second line of defense against a disrupted epithelium to systemically prevent the translocation of macromolecules and microbes [4]. It is unknown how ethanol-induced translocation of macromolecules into the intestinal mucosa affects intestinal microvascular endothelial cells.

An intact gut microbiome along with an optimal food supply (e.g., prebiotics) generates many beneficial metabolites for the host, including short chain fatty acids (SCFAs). Among these, butyrate serves as an energy source for epithelial cells, and supports intestinal epithelial barrier integrity and immune function [5]. In both humans [6] and rodents [7], ethanol-induced gut dysbiosis leads to the depletion of SCFAs, such as propionate and butyrate. In response to chronic-binge ethanol treatment in mice, depleted butyrate levels in the cecum coincide with decreased abundance of tight junctional proteins in the proximal colon, hepatic inflammation, oxidative stress, and steatosis [8]; these are all effects that can be mitigated by supplementing mice with a butyrate-targeting synbiotic or a butyrate pro-drug (tributyrin) [8]. This beneficial effect points to an important role of butyrate in protecting the colon and liver from injury induced by ethanol. However, the impact of ethanol and butyrate on the small intestinal epithelium and its secondary microvascular barrier function is unclear.

The intestinal tract comprises roughly 70% of the body’s immune capability, which is contained primarily within the mucosal layer [9], where the lamina propria contains a variety of immune cells. Dendritic cells (DCs) are professional antigen-presenting cells found within organized and diffuse lymphoid tissue of the small and large intestines where they serve to bridge innate and adaptive immunity. DCs’ interaction with microbes or microbial products like endotoxin can lead to proinflammatory cytokine production and T-cell activation, thus directing the type and degree of immune responses.

Following absorption through the intestinal epithelium, ethanol crosses the intestinal microvasculature prior to its entry into the portal vein and metabolism in the liver. Ethanol-induced gut dysbiosis allows for enteric opportunistic microbes and their byproducts to traverse the epithelium, enhancing the exposure of immune and nonimmune cells within the lamina propria to antigenic stimulation. Human trials have reported that alcohol consumption increases the severity of ulcerative colitis (UC) flare, which includes gastrointestinal symptoms of high severity [10]. 

Ethanol has a direct effect on vascular endothelium [11,12], but little is known about gut dysbiosis and acute ethanol exposure’s impact on intestinal microvasculature and intestinal dendritic cells. Given the key role butyrate plays in supporting the epithelial barrier and intestinal immune responses, here, we tested our hypothesis that butyrate supplementation during acute ethanol exposure would protect several components of intestinal integrity, including the gut microbiota’s composition, epithelial barrier, microvascular endothelium, and laminal propria dendritic cells. 

## 2. Results

### 2.1. Tributyrin Supplementation Mitigated Acute Antibiotic- and Ethanol-Induced Gut Microbial Disturbances

**Figure 1:**
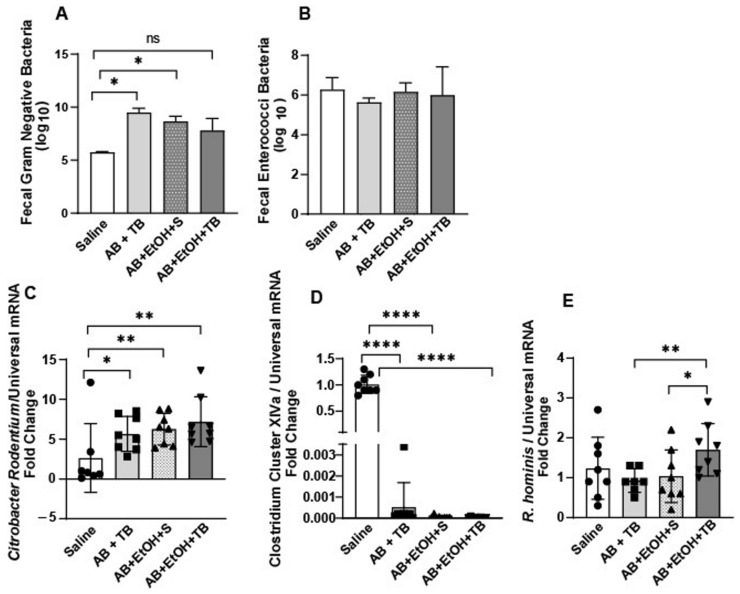
The effect of antibiotic, ethanol and tributyrin on select gut microbes. Mice received clindamycin subcutaneously (1.4 mg/kg body weight) and were randomized to receive either normal saline (100 µL) or tributyrin (100 µL; 2.5 mM) via oral gavage daily for 3 days. On day 4, mice were randomized to receive a single oral gavage of ethanol (5 gm/kg body weight) or maltose, along with either normal saline (100 µL) or tributyrin (100 µL; 5 mM) per previous supplement allocation. Fecal content from proximal colon was collected and diluted samples were plated on select agar (see Section 4) for (**A**) Gram-negative bacteria and (**B**) Enterococcus. Data are presented as mean log_10_CFU/g ± SEM. Cecum was excised and flash frozen at −80 °C until cecal content analysis by qRT-PCR (see Section 4) to assess the relative abundance of (**C**) *Citrobacter rodentium* species; (**D**) Clostridium Cluster XIVa; and (**E**) *Roseburia hominis* species. Data represent mean ± SEM. * *p* < 0.05; ** *p* < 0.001; **** *p* < 0.0001. ns: not significant.

Mice exposed to an antibiotic (clindamycin) for 3 days followed by a single gavage of ethanol exhibited overgrowth of Gram-negative bacteria in their colonic fecal content, but there was no effect on enterococcus growth (Figure 1A,B). Although Gram-negative bacterial growth was also induced in ethanol-exposed mice supplemented with tributyrin, a prodrug of butyrate [13], this was to a lesser extent and not statistically different from control mice, which were not exposed to antibiotics or ethanol (Figure 1A). *Citrobacter rodentium*, a Gram-negative mucosal pathogen of mice, was induced in all mice treated with antibiotics (Figure 1C). To determine the effect of tributyrin supplementation on the butyrate-generating capacity of the gut microbiota, we tested for the abundance of select butyrate-producing microbes. Clostridium Cluster XIVa (Clostridium coccoides group) is a predominant group of bacteria in the gut which produces beneficial metabolites, including butyrate and other SCFAs [14]. Mice exposed to antibiotics and/or ethanol exhibited a drastic reduction in the abundance of Clostridium Cluster XIVa (Figure 1D). *Roseburia hominis* is an anaerobic flagellated member of Clostridia Cluster XIVa, which produces butyrate and induces genes that are involved with promoting gut barrier function and innate immunity [15]. Interestingly, mice exposed to antibiotics and ethanol and co-supplemented with tributyrin had increased abundance of *R. hominis* compared to the other treatment groups (Figure 1E).

### 2.2. Tributyrin Supplementation Protected Small Intestinal Epithelial Integrity

**Figure 2:**
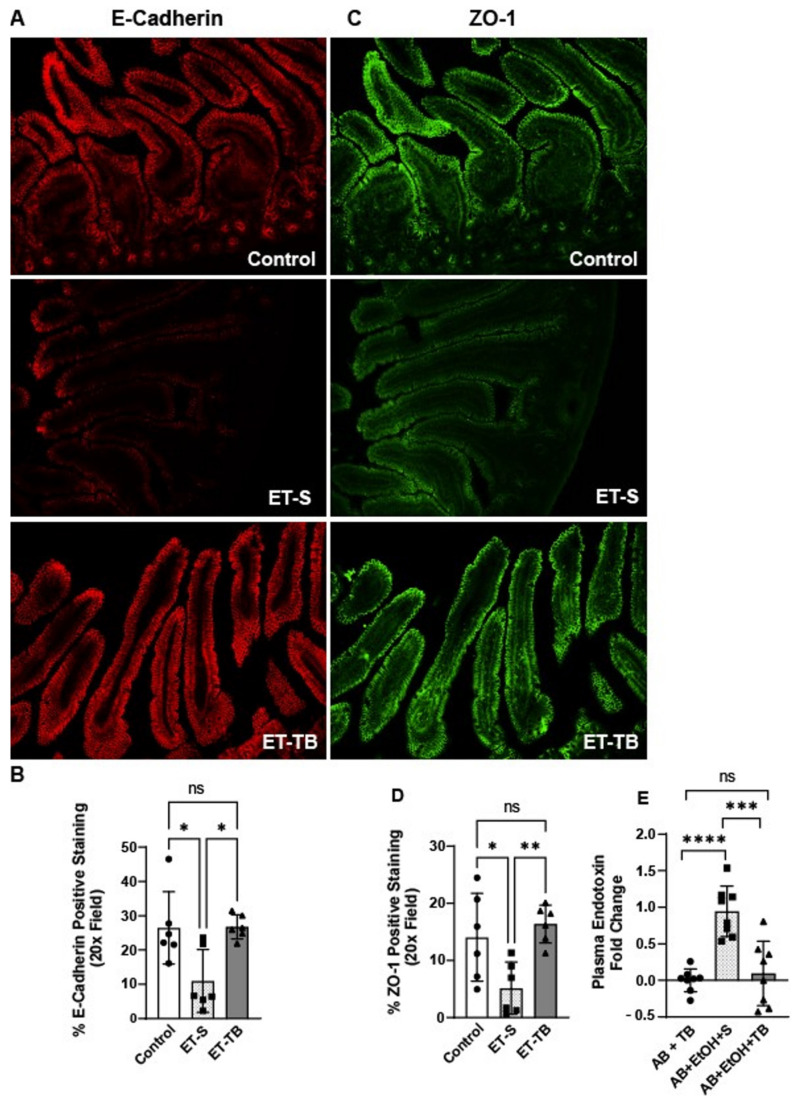
Epithelial tight junctional proteins in mouse jejunum and plasma endotoxin. Mice were randomized to receive normal saline (100 µL; control) or tributyrin (100 µL; 2.5 mM) three days prior to a single oral gavage of ethanol (5 gm/kg body weight) or maltose. Mice were euthanized 6 h after the acute ethanol and maltose gavage. Following euthanasia mouse jejunum was dissected, embedded in OCT and used for immunohistochemical analysis and semi-quantification of positive staining of (**A**,**B**) E-Cadherin and (**C**,**D**) zonulen-occludins-1 (ZO-1). All images were acquired using a 20X objective and are representative of a least replicate images captured per mouse in 6 mice per treatment group. In a separate experiment, mice received clindamycin subcutaneously (1.4 mg/kg body weight) and were randomized to receive either normal saline (100 µL) or tributyrin (100 µL; 2.5 mM) via oral gavage daily for 3 days. On day 4, mice were randomized to receive a single oral gavage of ethanol (5 gm/kg body weight) or maltose, along with either normal saline (100 µL) or tributyrin (100 µL; 5 mM) per previous supplement allocation. Mice were anesthetized and blood was drawn, and plasma was separated and tested for (**E**) endotoxin. Data represent 8 mice per treatment group and are presented as mean fold change ± SEM. * *p* < 0.05; ** *p* < 0.008; *** *p* < 0.0007; **** *p* < 0.0001. ns: not significant.

Ethanol exposure is known to alter the epithelial barrier of the proximal colon [16]. Here, we tested the effects of acute ethanol exposure on the epithelial barrier of the small intestine. Acute ethanol exposure caused disruption in tight junctional (TJ) protein (zonulen occluden-1 (ZO-1), E-Cadherin) localization in the jejunum of mice (Figure 2A–D), as demonstrated by immunohistochemistry (IHC). However, tributyrin supplementation maintained immune-reactive positive staining intensity of TJ similar to patterns seen in control mice (Figure 2B,D). Overgrowth of intestinal Gram-negative bacteria can disrupt intestinal barrier integrity and lead to increased endotoxin into systemic circulation [17]. Therefore, we tested plasma endotoxin levels amongst the treatment groups as a functional marker of an impaired intestinal barrier. As predicted, plasma endotoxin levels were induced in mice exposed to antibiotic and ethanol, while mice supplemented with tributyrin were protected against endotoxemia (Figure 2E).

### 2.3. Tributyrin Supplementation Attenuates Small Intestinal Microvascular Endothelium Activation

**Figure 3:**
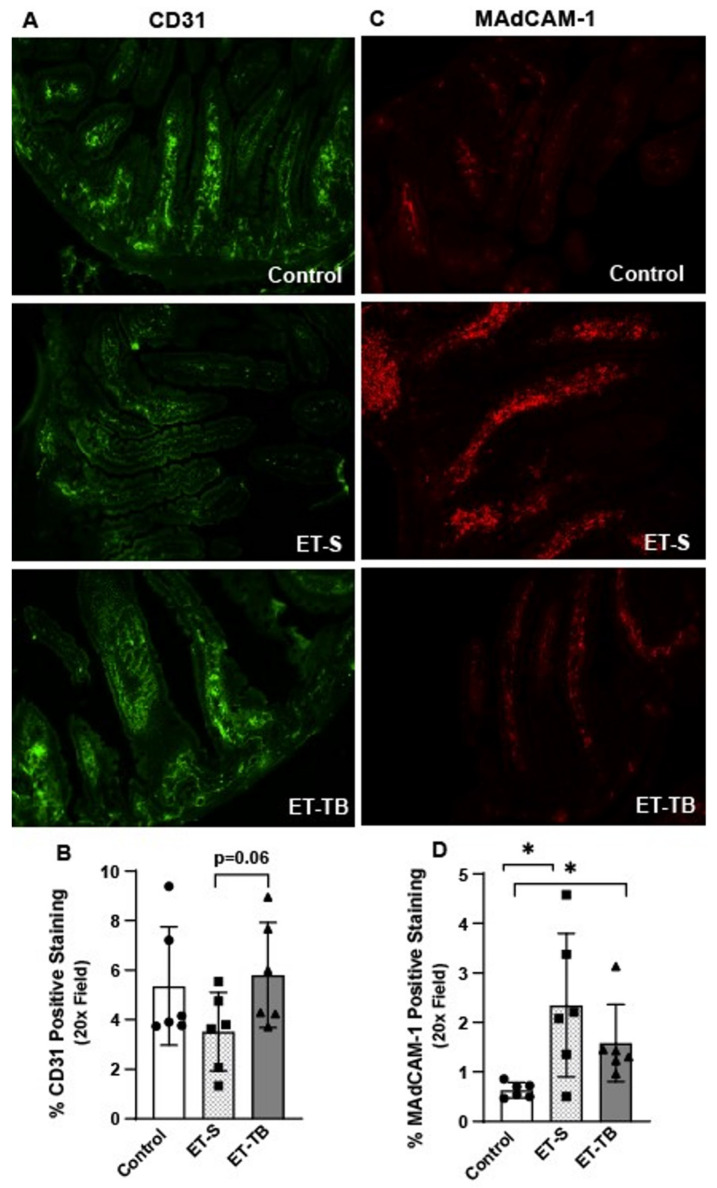
Effects of ethanol and tributyrin on small intestinal microvasculature. Mice were randomized to receive normal saline (100 µL; control) or tributyrin (100 µL; 2.5 mM) three days prior to a single oral gavage of ethanol (5 gm/kg body weight) or maltose. Mice were euthanized 6 h after the acute ethanol and maltose gavage. Following euthanasia mouse jejunum was dissected, embedded in OCT and used for immunohistochemical analysis and semi-quantification of positive staining (**A**,**B**) CD31 and (**C**,**D**) MAdCAM-1. All images were acquired using a 20X objective and are representative of a least replicate images captured per mouse in 6 mice per treatment group. Data represent means ± SEM. * *p* < 0.05.

Absorbed ethanol crosses intestinal capillaries prior to entering the portal vein on route to the liver, where it is primarily metabolized. CD31 or PECAM-1 (platelet endothelial cell-adhesion molecule-1) is a cell-adhesion molecule and signaling receptor that is highly expressed at the lateral borders of endothelial cell–cell junctions in confluent vascular beds [18]. Both localization and the relative amount of CD31 at paracellular junctions modulates endothelial junctional integrity [19]. We found that mice exposed only to ethanol had lower CD31 microvascular capillary density expression in the jejunum and that tributyrin supplementation corrected this disruption (Figure 3A,B). As ethanol exposure may impair both intestinal epithelial and microvascular endothelial barrier integrity, we tested for the effect of these impaired barriers and endotoxin translocation on small intestinal microvascular endothelium activation through analysis of mucosal vascular addressin cell-adhesion molecule-1 (MAdCAM-1) expression. MAdCAM-1 is constitutively expressed by intestinal endothelial cells, and its expression is upregulated on inflamed venules in chronic inflammatory diseases (e.g., inflammatory bowel disease) [20] and induced by TNF-α and IL-1β [20]. Here, we found that MAdCAM-1 was induced in mice exposed only to ethanol, and that tributyrin supplementation mitigated this effect (Figure 3C,D).

### 2.4. Tributyrin Supplementation during Acute Antibiotic and Ethanol Exposure Maintained a Tolerogenic Response in Lamina Propria Dendritic Cells

Conventional type 1 dendritic cells (cDC1) perform cross presentation of antigens to major histocompatibility complex (MHC) class I and initiate cytotoxic immune responses. Dendritic cells (DC) that are characterized by the expression of cluster of differentiation 103 (CD103; αE integrin) are located in the lamina propria, and CD103^+^ DC are known to promote tolerance. However, upon pathogen or pathogen byproduct (endotoxin) entry into the lamina propria, the tolerogenic response may be converted to a proinflammatory response in order to protect the host [21]. Lymphocyte Peyer’s patch adhesion molecule-1 (LPAM1; α4β7 integrin) is a receptor expressed on the cell surface of leukocytes and is involved in lymphocyte trafficking to sites of inflammation in intestinal tissues through its interaction with MAdCAM-1. As we found ethanol exposure in mice impaired the jejunal epithelial barrier and induced endotoxin translocation and MAdCAM-1 activation on small intestinal microvascular endothelium, we tested for the effect of these actions on DC in the small intestinal lamina propria. 

**Figure 4:**
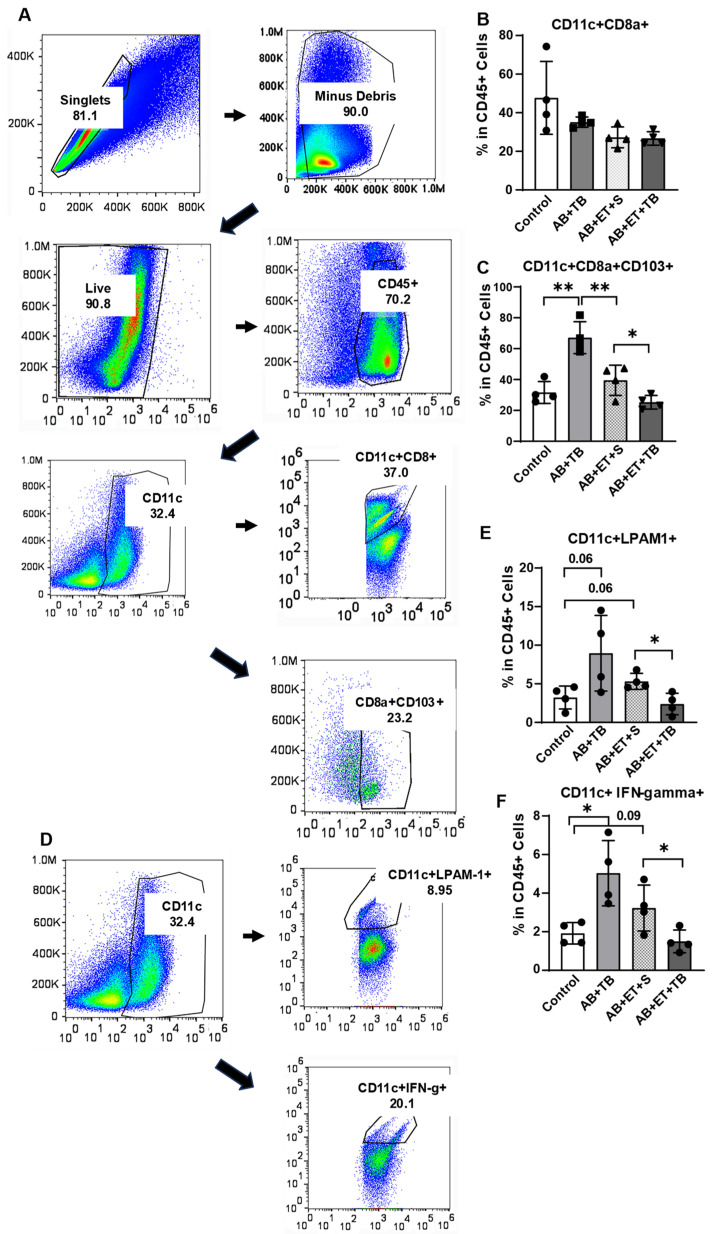
Effect of ethanol and tributyrin on dendritic cells in small intestinal lamina propria. Mice received clindamycin subcutaneously (1.4 mg/kg body weight) and were randomized to receive either normal saline (100 µL) or tributyrin (100 µL; 2.5 mM) via oral gavage daily for 3 days. On day 4, mice were randomized to receive a single oral gavage of ethanol (5 gm/kg body weight) or maltose, along with either normal saline (100 µL) or tributyrin (100 µL; 5 mM) per previous supplement allocation. Following euthanasia, the small intestine was dissected and immune single cell isolation from the lamina propria was performed (see Section 4). Following stimulation and fixation, cells were analyzed for expression of the following dendritic cell types via flow cytometry: (**B**) CD11c^+^CD8a^+^; (**C**) CD11c^+^CD8a^+^CD103^+^; (**E**) CD11c^+^LPAM1^+^; (**F**) CD11c^+^IFN-gamma^+^. Gating strategy is shown in panels (**A**,**D**). Percent of each cell type in CD45+ cells is presented as mean ± SEM with 4 mice per treatment group. * *p* < 0.05, ** *p* < 0.009.

Single cells harvested from the small intestinal lamina propria of mice were stained for surface receptors and analyzed by flow cytometry for expression of CD11c^+^CD8a^+^, and CD11c^+^CD8a^+^CD103^+^, CD11c+LPAM1^+^, and CD11c^+^IFN-gamma^+^ (Figure 4A,D). There were no differences in the CD11c^+^CD8a^+^ cells between the groups (Figure 4B). However, antibiotic exposure increased CD11c^+^CD8a^+^CD103^+^ positive cells in mice, which was diminished by ethanol exposure, and tributyrin supplementation during ethanol exposure further reduced CD11c^+^CD8a^+^CD103^+^ positive cells (Figure 4C). Additionally, tributyrin supplementation in ethanol exposed mice resulted in lower CD11c^+^LPAM1^+^ and CD11c^+^IFN-gamma^+^ positive cells compared to mice only treated with antibiotic/tributyrin or antibiotic/ethanol (Figure 4E,F). These data suggest that tributyrin supplementation maintained a tolerogenic DC response during ethanol exposure by protecting against an impaired jejunal epithelial barrier, endotoxin translocation, and MAdCAM-1 activation.

### 2.5. Sodium Butyrate Attenuated the Direct Effects of Ethanol and LPS on HIMEC Activation

**Figure 5:**
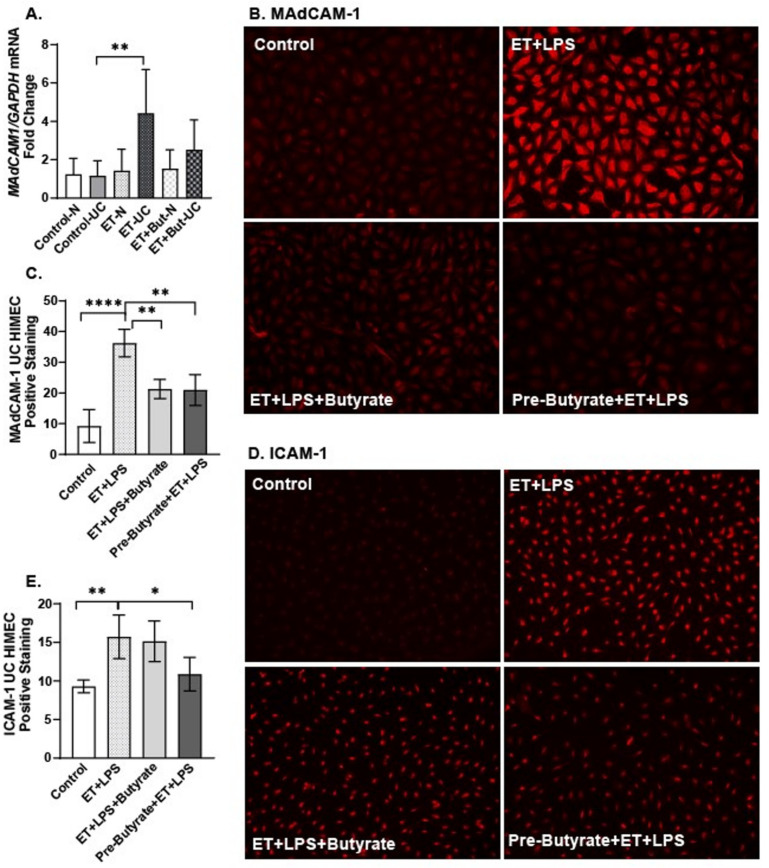
Direct effects of butyrate on ethanol plus LPS-induced HIMEC activation. Monolayers of primary HIMEC isolated from a patient without (N) and with ulcerative colitis (UC) were exposed to ethanol ± LPS and ±sodium butyrate for 60 min. In separate experiments, HIMEC monolayers were pre-treated with sodium butyrate for 24 h prior to and during exposure to ethanol ± LPS and ±sodium butyrate. Monolayers were harvested and used to prepare RNA for qRT-PCR of *MAdCAM-1* mRNA expression (**A**). Data represent mean ± SEM of relative fold change with 4 replicates per treatment group. HIMEC monolayers were also used for immunofluorescent analysis and semi-quantification of positive staining (red color) of (**B**,**C**) MAdCAM-1 and (**D**,**E**) ICAM-1. All images were acquired using a 20X objective and are representative of a least replicate images captured in 4 experimental replicates. * *p* < 0.05; ** *p* < 0.008; **** *p* < 0.001.

To test for the direct effect of ethanol and/or butyrate on intestinal microvascular endothelial cell activation, we utilized primary HIMEC monolayers derived from patients with ulcerative colitis (UC) and without ulcerative colitis (N) and evaluated alterations in MAdCAM-1 mRNA expression via qPCR and MAdCAM-1 and intracellular adhesion molecule (ICAM-1) protein expression via IHC. Ethanol challenge (20 mM, 1 h) significantly induced MAdCAM-1 mRNA expression only in HIMEC derived from a patient with UC (Figure 5A), and this expression trended lower in HIMEC-UC co-treated with ethanol and butyrate (20 µM), but the difference was not statistically significant. To test for protein expression of MAdCAM-1 and ICAM-1, and to model elements of the in vivo acute antibiotic/ethanol exposure in vitro, we treated HIMEC-UC with ethanol (20 mM) and LPS (1 µg/mL) for 1 h. HIMEC were also either co-treated with sodium butyrate (20 µM, 1 h) or pretreated with sodium butyrate for 24 h prior to and during ethanol and LPS treatment. 

We found HIMEC-UC monolayers demonstrated induced MAdCAM-1 and ICAM-1 protein expression when treated with ethanol and LPS (Figure 5B–E). Sodium butyrate pre-treatment and co-treatment attenuated MAdCAM-1 expression, and sodium butyrate pre-treatment attenuated ICAM-1 expression (Figure 5B–E).

## 3. Discussion

The present study investigated the role of butyrate supplementation on the effects of acute antibiotic and ethanol exposure on gut microbiota, small intestinal tight junctional proteins, circulating endotoxin, small intestinal lamina propria dendritic cells, and intestinal microvascular endothelial cell activation in mice. The data presented show for the first time that oral tributyrin supplementation induced a butyrate-generating gut commensal bacterial species, mitigated ethanol-induced overgrowth of Gram-negative bacteria, and protected the expression of proteins involved in maintaining the small intestinal epithelial barrier; additionally, it decreased endotoxemia, which coincided with promotion of immune tolerance of DCs in the small intestinal lamina propria and non-activation of intestinal microvascular endothelial cells.

Ethanol exposure is known to induce gut dysbiosis [2] and impair the intestinal epithelial barrier [8,16]. Gut microbiota depletion of beneficial and overabundance of potentially pathogenic microbes leads to reduced generation of key gut microbial metabolites. Butyrate, a SCFA derived from the fermentation of soluble fibers (e.g., prebiotics) in the distal gut, is essential to support the integrity of the large intestine [5], but little is known regarding butyrate’s ability to support the proximal small intestinal epithelial barrier during acute ethanol exposure. While the current study did not aim to fully interrogate the gut microbiome and metabolome, interventions were designed based on prior studies [16,22] and under the assumption that acute ethanol exposure and/or a broad-spectrum antibiotic would induce gut dysbiosis and skew gut microbiota-derived SCFAs. Tributyrin, a structured lipid and butyrate prodrug, is digested by pancreatic lipase to yield three butyrate molecules that can be absorbed or transported across the small and large intestinal epithelium [13]. Our prior studies have shown tributyrin co-supplementation in mice during acute and acute-on-chronic ethanol exposure [16,22] and antibiotic exposure [23] rescues tight junctional protein disassembly in mouse proximal colon. Here, we find that oral tributyrin co-supplementation during acute antibiotic and ethanol exposure induced the butyrate-generating species *R. hominis*, protected losses in TJ protein density (E. Cadherin, ZO-1) in jejunal epithelium and mitigated elevated circulating endotoxin levels to those of control mice. Whether these effects were directly related to tributyrin or indirectly through the induction of *R. hominis* deserves further investigation. 

Gut dysbiosis and epithelial barrier disruption trigger local inflammation. Lying just below the intestinal epithelial barrier is the lamina propria, which hosts many immune cells and a rich microvasculature. An impaired jejunal epithelium and elevated plasma endotoxin indicate translocated endotoxin would need to traverse the lamina propria and cross the microvasculature prior to entering the portal vein. This microvasculature is lined with endothelial cells which play a key role in mucosal immune homeostasis by regulating leukocyte migration from the intravascular to the interstitial space [4]. During inflammation, through the expression of cell-adhesion molecules (CAMs) and chemokine secretion, the intestinal microvasculature controls leukocyte flux. MAdCAM-1 is a gut-specific endothelial receptor which plays a key role in the recruitment of α4β7 integrin-expressing leukocytes into the mucosal lamina propria. Results from the current study show tributyrin supplementation prevented acute ethanol-induced MAdCAM-1 expression and trended towards protecting losses of CD31 (PECAM1) expression in mouse jejunum. Supporting microvascular endothelial cell–cell contacts and preventing microvascular endothelial cell activation with tributyrin supplementation could then influence immune cell responses within the lamina propria. 

SCFAs can regulate the activation, recruitment, and differentiation of immune cells and suppress inflammatory responses [24]. Conventional dendritic cells (cDCs) are found within the organized lymphoid tissue and the gut lamina propria, where they mediate tolerance by recognizing soluble antigens and commensal microbes, and they provide protective immune responses against pathogens [25]. Of interest is that butyrate has been shown to be a potent inducer of tolerogenic human DCs [24]. cDC1 can incorporate dying cells and produce inflammatory cytokines. In mice, lamina propria cDC1s are recognized by cell surface markers CD11c^+^CD8a^+^CD103^+^, and can acquire α4β7 (LPAM1), bind to MAdCAM-1 on intestinal microvascular endothelium, and home to the intestine [25]. We show that tributyrin-supplemented mice treated with antibiotics and ethanol had a lower percentage of CD11c^+^LPAM1^+^ and CD11c^+^IFN-gamma^+^ cells as well as CD11c^+^CD8a^+^CD103^+^ cells compared to mice only treated with ethanol. These data suggest that tributyrin’s protection of the intestinal epithelial barrier and subsequent reduction in antigen translocation into the lamina propria may also be appreciated with a blunted DC response, as compared to mice only exposed to ethanol. Interestingly, the percentage of cDC1 cells was higher in the antibiotic-/tributyrin-treated mice compared to both the antibiotic- and ethanol-treated mice, suggesting ethanol may influence cDC1 cells in the intestinal lamina propria either indirectly through its influence on gut microbiota or directly on immune and nonimmune cells in the lamina propria following its passive absorption. Further investigation is warranted to test how lamina propria DC may influence T-cell responses during acute and chronic ethanol exposures. Butyrate’s effect on gut mucosa is multi-faceted, and these complex interactions are still not completely understood. In summary, we believe that butyrate, and SCFAs in general, have complex immunomodulatory roles: there is a mechanical effect in augmenting intestinal barrier function; an anti-inflammatory signaling effect as a ligand for SCFA-sensing G-protein-coupled receptors (GPR43, GPR41, GPR109A); and an immune-stimulating effect, as butyrate modulates the innate and adaptive immune cell interactions within the intestinal mucosa. We have discussed these implications in detail in a previously published article [26]. 

Human data show that patients with UC are at higher risk of relapse when they consume alcohol [27]. It is thought this occurrence is related to ethanol’s negative effect on the gut microbiome and epithelial barrier disruption, leading to a proinflammatory milieu in the gut-associated lymphoid tissue. Higher intensity of inflammatory activity and worse outcomes are found when higher amounts of alcohol are consumed [28], and intestinal permeability is correlated with the location of diseased tissue [10]. Alcohol exposure has also been reported to increase the propensity of nonspecific enteral infections, including *Clostridioides difficile* infections, in patients with colitis, requiring frequent medical workups. These effects were consistent in an in vitro mouse model of colitis flare, which showed higher degree of inflammation [27,28]. However, the targets of ethanol-induced gut dysbiosis and an impaired intestinal barrier in inducing an inflammatory response is not fully understood. Here, we tested the direct effect of a physiologic concentration of ethanol on the activation of primary human intestinal microvascular endothelial cells (HIMEC) derived from subjects with and without UC as a potential link in mediating intestinal inflammation. An induction of MAdCAM-1 mRNA occurred only in HIMEC derived from a patient with UC (HIMEC-UC) following ethanol exposure. When modeling the potential effect of an impaired gut barrier using LPS and ethanol on HIMEC-UC, we found both MAdCAM-1 and ICAM-1 protein expression were upregulated. Interestingly, butyrate treatment mitigated this effect when provided before (MAdCAM-1, ICAM-1) and with (MAdCAM-1) ethanol and LPS. Antibodies that target α4β7 (LPAM1) are commonly used to treat IBD [25], and usually also favored due to their gut-specific effects; they also have a reported higher efficacy compared to anti-TNF therapy, specifically for moderate–severe UC [29]. Significant heterogeneity exists in response to the treatment among patients with UC, suggesting a role for yet-unknown modifying factors [30]. Whether patient-specific factors that impact gut microbiome and butyrate generation, such as diet, play a role in these responses needs further investigation. Although provision of postbiotic butyrate is not the simple answer to address the complex dysfunctional state in UC, microbial metabolite therapy is an important component of future therapeutic armamentarium [31]. Butyrate enema has been utilized for both UC and diversion colitis and has produced mixed results [32,33]. Similarly, many historic clinical studies have produced mixed results in regard to the utility of butyrate for gut inflammation; however, recent clinical trials have noted the potential of butyrate as an add-on therapy, as well as for the maintenance of remission [34,35]. Recent observational studies have noted lower fecal calprotectin levels and maintenance of remission in UC with microencapsulated butyrate [36]; however, there is a lack of substantial proof in terms of randomized controlled trials. Further understanding of this complex pathway can pave the way for exploration of personalized treatment for these patients based on their microbiome/metabolomic signature. 

## 4. Materials and Methods

### 4.1. Reagents

Primary antibodies were obtained from the following: anti-MADCAM-1 and anti-Zonulen Occludens-1 (ThemoFisher, Rockford, IL, USA), anti-HSC70 (Santa Cruz, Dallas, TX, USA), anti-CD31 (Bioss, Woburn, MA, USA), anti-E-Cadherin (Life Technologies Corp, Carlsbad, CA, USA), and anti-ICAM1 (Cell Signaling, Trask Lane Danvers, MA, USA). Antibodies for flow cytometry were purchased from the following: anti-CD8a-BUV615 (BD Biosciences, Franklin Lakes, NJ, USA), anti-CD11c-BV650, anti-CD103-BV785, and anti-INF-gamma-BUV737 (BioLegend, San Diego, CA, USA), anti-Lymphocyte Peyer’s patch adhesion molecule-1 (LPAM1-BV421 or integrin α4β7; BD Biosciences, Franklin Lakes, NJ, USA), and secondary antibodies Alexa Fluor 488 and 568 IgG (Invitrogen, Eugene, OR, USA). Clindamycin was purchased from Fresenius Kabi (Lake Zurich, IL, USA), and tributyrin, sodium butyrate, and lipopolysaccharide were obtained from Sigma-Aldrich (St. Louis, MO, USA). Primers used for quantitative real-time reverse transcription polymerase chain reaction (qRT-PCR) were synthesized by Integrated DNA Technologies (Coralville, IA, USA). 

### 4.2. Mouse Models

C57BL/6 female mice that were 10–12 weeks old (Jackson Laboratory, Bar Harbor, ME, USA) were housed in standard microisolator cages (2 mice per cage) and fed standard laboratory chow (rodent diet #2918; Teklad Envigo, West Lafayette, IN, USA). 

### 4.3. Ethanol Exposure and Tributyrin Supplementation

Two models of acute ethanol exposure were used to model a controlled acute dose. (1) A single oral gavage of ethanol (5 gm/kg body weight) or maltose was provided to the mice. The dosage was chosen based on a previously established acute ethanol exposure model which has been published by us previously, and this model has also been utilized as an established model by the National Institute on Alcohol Abuse and Alcoholism (NIAAA) to model for acute binge drinking [37]. This model results in systemic effect of ethanol after 6 h of ingestion. Mice were randomized to receive normal saline (100 µL; control) or tributyrin (100 µL; 2.5 mM) three days prior to the ethanol gavage that contained normal saline or tributyrin (5 mM) as part of the ethanol gavage per previous supplement allocation. Mice were euthanized 6 h after the acute ethanol and maltose challenge. Control mice received saline gavage to control the element of stress-related microbiome changes. (2) Mice received clindamycin subcutaneously (1.4 mg/kg body weight) for 3 days to induce disruption in gut microbiota. On day 4, a single oral gavage of ethanol (5 gm/kg body weight) or maltose was provided to mice. Mice were randomized to receive normal saline (100 µL) or tributyrin (100 µL; 2.5 mM) concurrently with the clindamycin treatment. Tributyrin (5 mM) or normal saline was provided in the oral ethanol gavage per previous supplement allocation. Mice were euthanized 6 h after the acute ethanol challenge. Mice were anesthetized and blood was collected from the posterior vena cava and plasma was separated and stored at −80 °C. Intestinal sections (jejunum, cecum) were dissected, and the cecum and jejunum were snap frozen in liquid nitrogen, and the jejunum was also stored for RNA extraction (RNALater, Ambion, Austin, TX, USA) or frozen for tissue sectioning and histological analysis (optimal cutting temperature compound (OCT), Sakura Finetek, Torrence, CA, USA). Fecal pellets were expelled from the proximal colon and immediately plated for bacterial growth. 

### 4.4. Fecal Plating

Select agar plating was performed on fresh fecal content isolated from the mouse colon during euthanasia. Plating was performed as previously described [38]. Briefly, following weighing, fecal content was suspended at 10 mg/µL, serially diluted, and plated for selection and differentiation of Gram-negative bacteria (Difco MacConkey Agar, BD, Franklin Lakes, NJ, USA) and enterococci (BBL Enterococcosel Agar, BD, Franklin Lakes, NJ, USA). The plates were then incubated at 37 °C in an aerobic environment and counted 24–48 h following plating. 

### 4.5. Fecal qRT-PCR

Mouse cecal content underwent total bacterial genomic DNA (gDNA) isolation (Quick-DNA Fecal/Microbe MiniPrep Kit, Zymo Research, Irvine, CA, USA) and quantification. Absolute levels of select fecal bacterial strains were determined with gDNA (5 ng) amplification via real-time PCR (QuantStudio 5 analyzer and PowerUp SYBR Green Master Mix, Applied Biosystems, Waltham, MA, USA) with bacterial primer sequences listed in Section 4.6. The comparative threshold (CT) method was used to determine relative abundance of the target bacteria to the total universal bacteria. Data are presented as fold changes relative to saline-treated mice (controls). 

### 4.6. Tissue qRT-PCR

Total RNA from HIMEC was extracted (RNeasy Plus Universal Mini Kit, Qiagen Hilden, Germany), quantified (NanoDrop ND1000 Spectrophotometer), reverse transcribed (SuperScript IV VILO, Invitrogen, Waltham, MA, USA), and amplified via real-time PCR (QuantStudio 5 analyzer and PowerUp SYBR Green Master Mix, Applied Biosystems, Waltham, MA, USA) with the following primer sequences: Mucosal addressin cell-adhesion molecule 1 (MAdCAM-1): F: 5′-AGG TTT ATT TGC CAA AGC CTC-3′ R: 5′-CCC CTG TGA AAG CAA AAT AGC-3′; glyceraldehyde 3-phosphate (GAPDH): F: 5′-CGT CTT CAC CAC CAT GGA GA-3′; R: 5′-CGG CCA TCA CGC CAC AGT TT-3′. The comparative threshold (CT) method was used to determine the relative expression of the target gene (MADCAM-1) to the housekeeping gene (GAPDH). Data are presented as fold changes relative to saline-treated mice (controls).

### 4.7. Endotoxin Assay

Careful techniques were used to avoid microbial or endotoxin contamination during blood collection and experimental procedures. All materials including reagents, pipette tips, pipettes, and 96-well plates were endotoxin-free. The glass tubes for samples were incubated at 250 °C for 40 min to ensure they were endotoxin-free. Plasma samples were diluted 10^4^ times with 10 mM MgCl_2_ in LAL Reagent Water Lonza (Walkersville, MD, USA). Kinetic Assay was performed with Limulus Amebocyte Lysate (LAL) Kinetic-QCL kit (Lonza, Walkersville, MD, USA). The mean time and standard curve were performed with *Escherichia coli* O55:B5 Endotoxin (Lonza, Walkersville, MD, USA). SpectraMax^®^ Microplate Readers and SoftMax^®^ Pro 7.1.2 GxP Software Molecular Devices (San Jose, CA, USA) were used for reading the plate and calculating the endotoxin concentration in samples. Data are presented as endotoxin fold change relative to mice exposed to the antibiotic and tributyrin. 

### 4.8. Lamina Propria Immune Cell Isolation and Activation

Immediately following euthanasia, the small intestinal tract (pylorus to distal ileum) was dissected, and single immune cells were isolated from the lamina propria, per the detailed procedure described in Qiu et al. [39]. Immediately following isolation, lamina propria immune cells were stimulated with phorbol 12-myristate 13-acetate (PMA, 10 ng/mL)/ionomycin (1 µM) (MilliporeSigma, Darmstadt, Germany) for 4 h and in the presence of 2 µM monensin (Calbiochem, San Diego, CA, USA) during the last 2 h of stimulation. Cells were washed and harvested for staining and analysis via flow cytometry. 

### 4.9. Flow Cytometry

Following immune cell activation, single cells were stained with Live and Dead Fix Blue (Invitrogen, Carlsbad, CA, USA) for 30 min in the dark. After washing the cells with staining buffer, cells were fixed and permeabilized (True-Nuclear Transcription Factor Buffer Set, Bioligand, San Diego, CA, USA). Cells were blocked for 30 min to reduce nonspecific staining (FcR Blocking reagent, MIltenyi Biotec, Gaithersburg, MD, USA) and then stained with fluorescence-conjugated antibodies anti-CD8a-BUV615, anti-CD11c-BV650, anti-CD103-BV785, anti-INF-gamma-BUV737, and anti-LPAM-1-BV421 for one hour. Cells were washed 3 times and cells were acquired using a Sonny ID 7000 Spectral Cell Analyzer (San Jose, CA, USA).

### 4.10. Immunohistochemistry (IHC)

Jejunal sections frozen in OTC were used for immunostaining of select proteins of the intestinal epithelial tight junctional protein complex (E-Cadherin and Zonulen occludens-1 (ZO-1); immunostaining of endothelial cells (Platelet endothelial cell-adhesion molecule-1 (PECAM-1) or CD31; and MADCAM-1, an endothelial cell-adhesion molecule, as previously described [38]. HIMEC were used for immunostaining for MADCAM-1 and ICAM-1 (CD54), a cell surface glycoprotein and an adhesion receptor. Sections incubated with secondary antibodies only (negative control) did not reveal specific immunostaining versus those incubated with primary antibodies. A single investigator blinded to treatment groups viewed all slides. Three images were obtained per tissue section with 4–6 mice per treatment group. Semi-quantification of positive staining was performed using Image Pro Plus software v10 (Media Cybernetics, Silver Spring, MD, USA). Images were captured using a fluorescence microscope (Keyence B2-X810, Istasca, IL, USA).

### 4.11. Human Intestinal Microvascular Endothelial Cells (HIMECs) Culturing 

In vitro studies were conducted with primary HIMECs derived from colon sections, as previously described [40], from subjects that had histologically normal tissue and UC. Isolated PECAM-1 (CD31)-positive HIMECs were cultured. Monolayers were treated for 60 min with ±LPS (1 µg/mL), ±sodium butyrate (20 µM), and/or ±20 mM ethanol (a physiologic reference level for blood alcohol when judgment and coordination are clinically impaired). Cells were then utilized for immunohistochemical analysis or harvested for RNA extraction and qPCR analysis. HIMEC experiments included at least 3 replicates per treatment for each experiment, and each experiment was repeated 4–6 times utilizing cells within 4–10 cell passages.

### 4.12. Statistical Analysis

Data are presented as the mean ± standard error of the mean (SEM). A total of 4–6 mice were included per treatment groups and 4–6 replications were included for cell culture experiments with power based on plasma endotoxin level as primary outcome indicating 4–6 mice per group required to determine statistical significance of *p* < 0.05 at 80% power. The parametric analysis of 2 groups utilized student’s *t*-test, and comparison of multiple groups utilized analysis of variance, with Tukey’s post hoc test for multiple comparisons. Statistical significance was defined as *p* < 0.05. Prism software version 10.1.2 (324) (GraphPad Software, San Diego, CA, USA) was used to perform the analyses.

## 5. Conclusions

Ethanol has complex multi-level effects on the gastrointestinal mucosal system. Here, we show that ethanol negatively altered the gut microbiome within the intestinal lumen, and impaired intestinal epithelial barrier integrity through its disruption of the small intestinal tight junctional protein complex; this allowed the transmigration of endotoxin into systemic circulation. Translocated endotoxin reaches the lamina propria where it can interact with immune cells which are homed to the gut mucosa. We show ethanol-modulated intestinal microvascular endothelial cells through activation of the MADCAM-1 and ICAM-1 pathway, which may then induce the recruitment of immune cells from systemic circulation. We found that butyrate supplementation mitigated each of these multi-level detrimental effects of ethanol on the intestinal mucosal layer. We implemented a novel combination of in vivo experiment, utilizing mouse models of acute ethanol exposure, and in vitro techniques, utilizing human endothelial cell monolayers to study the direct effects of ethanol; however, there are several limitations which were beyond the scope of our study. Specifically, in vivo the interaction of ethanol with human microvascular endothelial cells is further modified by intestinal epithelial cells and immune cells and secreted cytokines within the lamina propria. In the current paper, we were not able to explore this interaction in relation to the endothelial and epithelial cells. Further studies are needed to completely understand the complex interaction between ethanol, the gut microbiome, and gut mucosal immune components. A deeper understanding of these interactions could help develop patient-specific microbiome reprogramming approaches to be used in conjunction with other therapy in patients with an inflammatory disorder such as ulcerative colitis.

## Data Availability

Data are contained within the article.
